# BRF2 is mediated by microRNA-409-3p and promotes invasion and metastasis of HCC through the Wnt/β-catenin pathway

**DOI:** 10.1186/s12935-023-02893-y

**Published:** 2023-03-16

**Authors:** Jian-Hua Chang, Bo-Wen Xu, Di Shen, Wei Zhao, Yue Wang, Jia-liang Liu, Guang-Xiao Meng, Guang-Zhen Li, Zong-Li Zhang

**Affiliations:** 1 Department of General Surgery, Qilu Hospital, Cheeloo College of Medicine, Shandong University, No.107 Wenhua West Road, Lixia District, Jinan, 250012 Shandong China; 2 Department of General Surgery, Gansu Province Hospital, Lanzhou, 730000 GanSu Province China; 3 Department of Hepatobiliary Surgery, National Cancer Center/National Clinical Research Center for Cancer/Cancer Hospital, Chinese Academy of Medical Sciences and Peking Union Medical College, Beijing, 100021 China; 4 Department of Obstetrics and Gynecology, Shandong Provincial Maternal and Child Health Care Hospital, Cheeloo College of Medicine, Shandong University, Jinan, 250012 Shandong Province China

**Keywords:** BRF2, miR-409-3p, Invasion, Hepatocellular carcinoma, Metastasis, Wnt/β-catenin

## Abstract

Hepatocellular carcinoma (HCC) is one of the most common cancers worldwide. Its invasiveness and ability to metastasize contributes to an extremely high patient mortality. However, the molecular mechanisms that underlie the characteristics of HCC progression are not well understood. BRF2 has been shown to be an oncogene in a number of tumors; however, its role in HCC has not yet been thoroughly examined. In this study, we identified and validated BRF2 as an oncogene in HCC, providing a new insight into HCC pathogenesis and therapeutic possibilities. We showed that BRF2 expression was significantly upregulated in HCC cell lines and tissues, while BRF2 depletion suppressed HCC metastasis and invasion. We then examined the upstream regulation of BRF2 and identified miR-409-3p as being predicted to bind to the 3′ UTR of BRF2. We used a luciferase activity assay and functional verification to show that BRF2 is downregulated by miR-409-3p. Finally, we used bioinformatic analysis to show that BRF2 may be related to early HCC development through the Wnt/β-catenin signaling pathway.

**Supplementary Information:**

The online version contains supplementary material available at 10.1186/s12935-023-02893-y.

## Introduction

Hepatocellular carcinoma (HCC) is the most common form of primary liver cancer, and the fifth most common malignancy in the world [1–3]. Over recent years, tremendous progress has been made in the diagnosis and management of HCC (through surgery, targeted therapy and immunotherapy) [3]; however, the 5-year survival rate of patients with HCC remains poor [4]. Consequently, novel and effective biomarkers for the diagnosis and treatment of HCC are urgently required.

RNA polymerase III (RNA Pol III) is a eukaryotic multi-subunit DNA-dependent enzyme that transcribes short, untranslated RNAs involved in fundamental cellular processes [5]. TFIIIB -related factor 1 (BRF1) and TFIIIB-related factor 2 (BRF2) are key subunits of a transcription factor required for the accurate recruitment and initiation of RNA Pol III [6]. Currently, there is no evidence linking changes in the expression of BRF1 to human cancers [6]; however, BRF2 has been shown to be overexpressed in many malignancies and plays a vital role in carcinogenesis [7, 8]. Despite this, the role of BRF2 in HCC remains largely unknown. MicroRNAs (miRNAs) are short, endogenous, single-stranded, non-coding RNA transcripts. MiRNAs can bind to the 3′ untranslated region (3′ UTR) of mRNA transcripts, resulting in post-transcriptional changes to gene expression [6, 7]. In this study, we used the RNA Target Scan database to identify miRNAs that can bind to the 3′ UTR of BRF2, and determined that miR-409-3p is predicted to bind to the 3′ UTR of BRF2 with high affinity. MiR-409-3p has been observed to be downregulated in many human tumors, including breast cancer [8, 9], bladder cancer [10], and colorectal cancer [11, 12]. MiRNAs have previously been shown to be useful as biomarkers, and some have been shown to act as tumor suppressors in lung cancer [13]. However, the role of miR-409-3p in HCC remains unknown.

In this study, we observed higher expression levels of BRF2 in HCC tissues and cell lines compared with noncancerous tissues and cell lines. Additionally, we observed that high levels of BRF2 were positively associated with invasion and migration of HCC cells, while levels of miR-409-3p were decreased in HCC tissues and cell lines. Importantly, miR-409-3p was negatively associated with the development of HCC, through its role as an inhibitor of BRF2 expression. Furthermore, transcriptome sequencing analysis showed that BRF2 affects the Wnt pathway. Collectively, our findings firmly establish that BRF2 expression is regulated by miR-409-3p, and can facilitate invasion and metastasis of HCC cells through the Wnt/β-catenin pathway.

## Materials and methods

### Human hepatocellular carcinoma specimens

Forty-five samples of human hepatocellular carcinoma tissue and paired paraneoplastic tissues were obtained from patients who underwent surgery at Qilu Hospital of Shandong University between June 2011 and June 2021. The tissues were removed, and stored in liquid nitrogen. Written informed consent was obtained from each patient and family members in accordance with the requirements of the Ethics Committee of Qilu Hospital, Shandong University.

### Cells and cell culture

The HCC cell lines Huh-7, HMCC-97H, SMMC-7721, HepG2, and Hep3B were kind gifts from Zhao-ru Dong (Department of Hepatobiliary Surgery, Qilu Hospital, Shandong University). The HCC cell lines were cultured in Dulbecco’s modified eagle medium (DMEM) (Gibco, Beijing, China) supplemented with 10% fetal bovine serum (FBS) (Gibco, Beijing, China) in a humidified atmosphere at 37 °C and 5% CO_2_.

### Oligonucleotide and plasmid transfection

The negative control (NC), BRF2 small interfering (si) RNA, BRF2 overexpression plasmid (OE), miR‐409‐3p inhibitor, and miR‐409‐3p mimics were all purchased from Gene Pharma (Shanghai, China). LGK974 was obtained from MedChemExpress (Monmouth Junction, NJ, USA). Huh-7 cells were plated into 6‑well plates at a density of 1 × 10^5^ cells per well and cultured for 24 h until they reached 80% confluency. The medium was then replaced with 1.5 mL fresh DMEM without antibiotics or antimycotics. Transfections were performed by mixing Lipofectamine 2000 (Invitrogen, Rockford, IL, USA), 100 pmol siRNA (NC or BRF2), miR‐409‐3p inhibitor, or miR‐409‐3p mimic, and OptiMEM (Thermo Fisher Scientific, Rockford, IL, USA). This mixture was incubated with Huh-7 cells for 6 h. The medium was then discarded and replaced with DMEM with 10% FBS. The targeting sequences were as follows: BRF2 siRNA, GCACUUACAUGCAGAUAGUTT; siRNA‑NC, UUCUCCGAACGUGUCACGUTT; miR‐409‐3p inhibitor, AGGGGUUCACCGAGCAACAUUC; miR‐409‐3p mimic, GAAUGUUGCUCGGUGAACCCCUGGGUUCACCGAGCAACAUUCUU. Cells were collected for protein and RNA analysis 12 h after transfection.

### Real-Time Quantitative Polymerase Chain Reaction (RT‑qPCR)

We used Triazole reagent (Invitrogen) to extract total RNA from cells or human tissues, according to the manufacturer’s instructions. The concentration of RNA was measured using a NanoDrop ultra-violet spectrometer (Thermo Fisher Scientific). The cDNA was reverse‑transcribed from the mRNA using the Prime Script RT reagent kit (TaKaRa, Tokyo, Japan). Real-time PCR was performed using Fast SYBR Green Master Mix (Applied Biosystems, Rockford, IL USA) with 3 sub-well replicates. Thermocycling conditions were chosen according to the manufacturer’s protocol. All results were normalized to GAPDH mRNA. The primers for GAPDH and BRF2 were: GAPDH, forward GCACCGTCAAGGCTGAGAAC and reverse TGGTGAAGACGCCAGTGGA; BRF2 forward as mentioned above. Relative gene expression was then analyzed using the ΔΔC_q_ method. Each experiment was performed at least three times.

### Western blotting

Western blotting was performed as described previously [14]. Total protein in cells and tissues was extracted with RIPA lysis buffer (Thermo Fisher), and the protein concentration was determined using the Pierce bicinchoninic acid Protein Assay kit (Thermo Fisher Scientific). Proteins were separated by 10% SDS-PAGE in running buffer (Servicebio, Wuhan, China), and then were transferred on to polyvinylidene difluoride membranes (Millipore Corporation, Bedford, MA, USA) in transfer buffer (Servicebio). The membranes were blocked in 5% non-fat milk, then incubated with primary antibodies overnight at 4 °C. The primary antibodies were: rabbit anti‑E‑cadherin (ab40772, 1:1000, Abcam, Cambridge, UK), rabbit anti‑N‑cadherin (ab18203, 1:1000, Abcam), anti-β-catenin (ab32572,1:1000, Abcam), anti-APC (ab40778, 1:2,000, Abcam), anti-GSK3 beta (ab32391, 1:2000, Abcam), anti-Axin2 (ab109307, 1:1000, Abcam), anti-CK1 (ab302638, 1:1000, Abcam), anti-wnt5A (ab229200, 1:1000, Abcam), mouse anti‑GAPDH (sc-47724, 1:1000, Santa Cruz Biotechnology Inc, Santa Cruz, CA, USA), mouse anti-BRF2 (sc-390312,1:1000, Santa Cruz Biotechnology Inc). GAPDH served as an internal control. The membranes were washed and incubated with the secondary antibody goat anti-rabbit IgG H&L (ab150113, 1:2,000, Abcam) or goat anti-mouse IgG H&L (ab6789, 1:2,000, Abcam) at 37 °C for 2 h, then washed with 0.9% TBST three times. The membranes were then incubated with the enhanced chemiluminescence kit (Millipore Corporation, Bedford, MA, USA) for visualization.

### Wound‑healing assay

Transfected cells were trypsinized and passaged in six-well plates at a density of 1 × 10^5^ cells per well. When cells reached 100% confluence, wounds were made in the monolayer with a sterile 1 mL pipette tip. The cells were washed three times using PBS to clear detached cells, followed by incubation with DMEM without FBS at 37 °C. The healing process was observed and the average distance between cells was calculated using ImageJ software (version 6.0).

### Transwell invasion and migration assay

For the invasion assay, we precoated the bottom of the upper chambers of transwell plates (8 μm, Corning Costar, MA, USA) with Matrigel (BD Biosciences, San Jose, CA), diluted at a ratio of 1:8. Cells in serum-free medium were seeded on to the filter of the upper chambers, and DMEM containing 10% FBS was added to the lower chambers. The plates were then incubated for 48 h. For the migration assay, the plates were not precoated in Matrigel. After 48 h, non-invading cells were gently removed with a cotton swab. The invading cells were fixed in 4% paraformaldehyde for 15 min, then stained with 0.1% crystal violet solution for 30 min. The images were taken from five randomly selected areas under the microscope.

### Luciferase activity analysis

293 T Cells and Smmc-7721 Cells were transfected with 200 ng pmirGLO plasmid using Lipofectamine 2000 (Invitrogen) with NC mimic + BRF2-WT, hsa-miR-409-3p mimics + BRF2-WT, NC mimic + BRF2 mut, or hsa-miR-409-3p mimics + BRF2-mut. The renilla luciferase reporter vector pRL-TK was used as an internal control. After 48 h following transfection, firefly and renilla luciferase activities were sequentially detected by the Dual Luciferase Reporter Assay system (Promega, Madison, WI, USA).

### Animal study

The animals were used according to an experimental protocol approved by the Medical Experimental Animal Care Commission of Shandong University. For the in vivo metastasis assay, 4–5-week-old female immunodeficient mice (nude mice; 5 per group) were injected were injected with 1 × 10^6^ cells that were transfected with either NC lentivirus (control) or BRF2 knockdown lentivirus (sh-BRF2) through the lateral tail vein. The mice were then executed six weeks later according to standard procedure. The livers and lungs were collected and fixed in 10% buffered formalin. The lung samples were embedded in paraffin, sectioned, and stained with hematoxylin and eosin (H&E).

### Immunohistochemistry

Paraffin sections of tumor tissues were cut at a thickness of 5 μm. Sections were incubated with the primary antibody anti-CK8 (ab53280, 1:200, Abcam) at 4 °C overnight. Sections were incubated with HRP‐polymer-conjugated secondary antibodies after washing with phosphate‐buffered saline, and then they were immunostained using a DAB plus kit (ZSGB-Bio, Beijing,China).

### Statistical analysis

Data analysis was performed by SPSS statistical software (version 11.5). Numerical data are shown as the mean ± SEM (standard error of mean). Comparison of categorical variables was calculated using Fisher’s exact test or χ^2^ test. Two-tailed tests were used for statistical analysis. Statistical significance was considered to be P < 0.05.

## Results

### BRF2 expression levels were upregulated in HCC tissues and cells

**Fig. 1:**
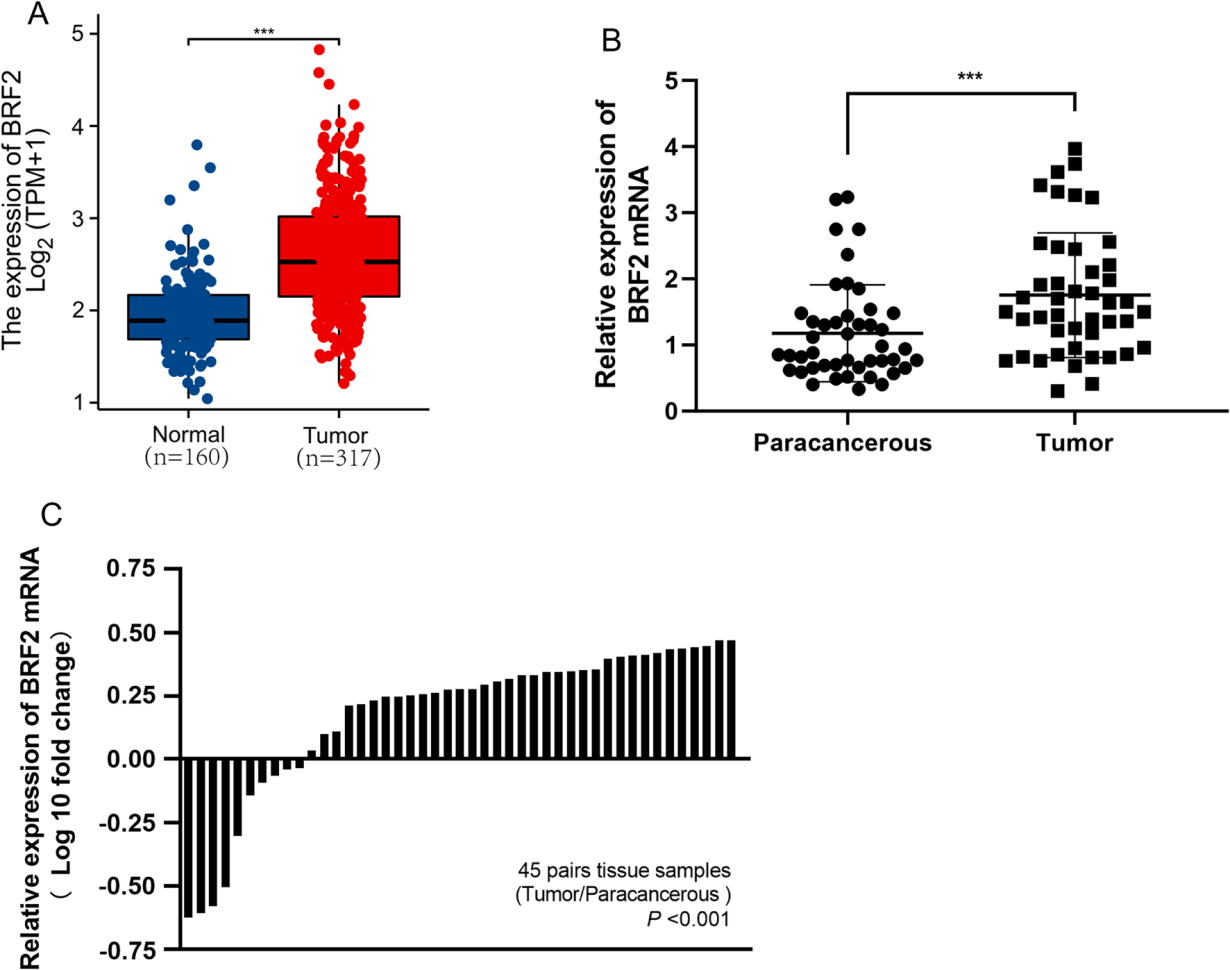
BRF2 is overexpressed in hepatocellular carcinoma. **A** BRF2 expression in tumor (317) and normal tissues (160) based on TCGA and the Genotype-Tissue Expression (GTEx) database. ∗  ∗  ∗ , P < 0.001. **B** RT-qPCR data showing the expression of BRF2 in 45 pairs of hepatocellular carcinoma and adjacent, noncancerous tissues. **C** Paired comparison of BRF2 expression levels between hepatocellular carcinoma and corresponding normal tissues (Tumor/Paracancerous)

**Fig. 2:**
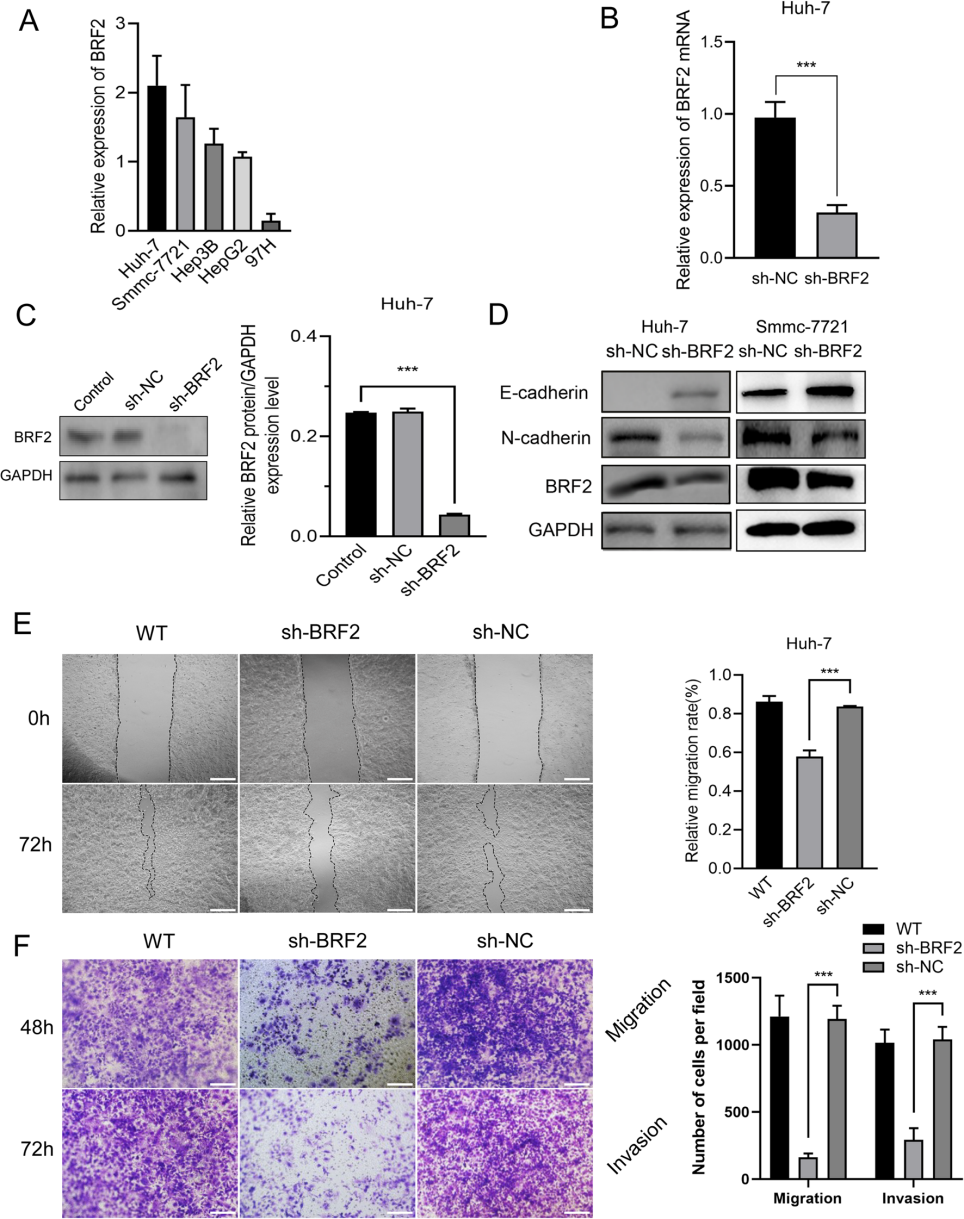
BRF2 knockdown inhibits the invasion and metastasis of liver cancer Huh-7 cells. **A** The levels of BRF2 mRNA in five types of liver cancer cells were measured by RT-qPCR. **B** The levels of BRF2 mRNA in Huh-7 cells after lentiviral infection with BRF2 short-hairpin RNA (sh-BRF2) or control short-hairpin RNA (sh-NC) were measured by RT-qPCR. ∗  ∗  ∗ , P < 0.001. **C** Left: Representative western blot data showing BRF2 levels in Huh-7 cells after lentiviral infection with sh-BRF2 and sh-NC. Right: BRF2 levels are presented as the mean ± standard deviation (SD); n = 3. ∗  ∗  ∗ , P < 0.001 compared with the control group. **D** Representative western blot data showing E-cadherin and N-cadherin in Huh-7 cells after lentiviral infection with sh-BRF2 and sh-NC. **E** Invasion and migration of Huh-7 cells after lentiviral infection with sh-BRF2 and sh-NC were measured by wound-healing assay. Left: Representative images at 100 × magnification; scale bar, 100 µm. Right: Quantification of wound-healing assay. WT, Wild type. ∗  ∗  ∗ , P < 0.001, sh-NC vs sh-BRF2 group. **F** Left: Representative images of transwell migration and invasion assays in Huh-7 cells after lentiviral infection with sh-BRF2 and sh-NC. Magnification, 200 × ; scale bar, 50 µm. Right, Quantification of transwell migration and invasion assays. n = 3. *WT* Wild type. ∗  ∗  ∗ , P < 0.001. sh-NC vs sh-BRF2 group

Relative levels of BRF2 mRNA were significantly increased in liver cancer tissues compared with normal liver tissues according to bioinformatic analysis of several databases (Fig. 1A). Consistently, we observed increased BRF2 mRNA levels in liver cancer tissues compared with the adjacent normal tissues in clinical specimens (Fig. 1B, 1C). BRF2 expression in Huh-7, HMCC-97H, SMMC-7721, HepG2, Hep3B, and normal liver cells was monitored via RT‑qPCR. The highest BRF2 expression was observed in Huh-7 cells and SMMC-7721 cells (Fig. 2A).

### BRF2 promotes migration and invasion in vitro

In order to study the characteristics of BRF2 in live cancer cells, siRNA targeting BRF2 or a negative control (si-NC) was transfected into Huh-7 and SMMC-7721 cells. Following BRF2 depletion, the relative mRNA (Fig. 2B) and protein (Fig. 2C) levels of BRF2 were significantly lower than in the control group. Additionally, we monitored the relative migration ability of the two groups using a wound-healing assay and observed a considerable reduction in migration in the BRF2 siRNA-treated group compared with the si-NC treated group (Fig. 2E). Furthermore, we depleted BRF2 using shRNA in Huh-7 and SMMC-7721 cells and found that BRF2 knockdown reduced cell invasion and migration (Fig. 2F). Reducing BRF2 expression also reduced the level of N‑cadherin and increased the level of E‑cadherin in cells according to western blot analysis (Fig. 2D).

### MiR-409-3p suppresses BRF2 expression

**Fig. 3:**
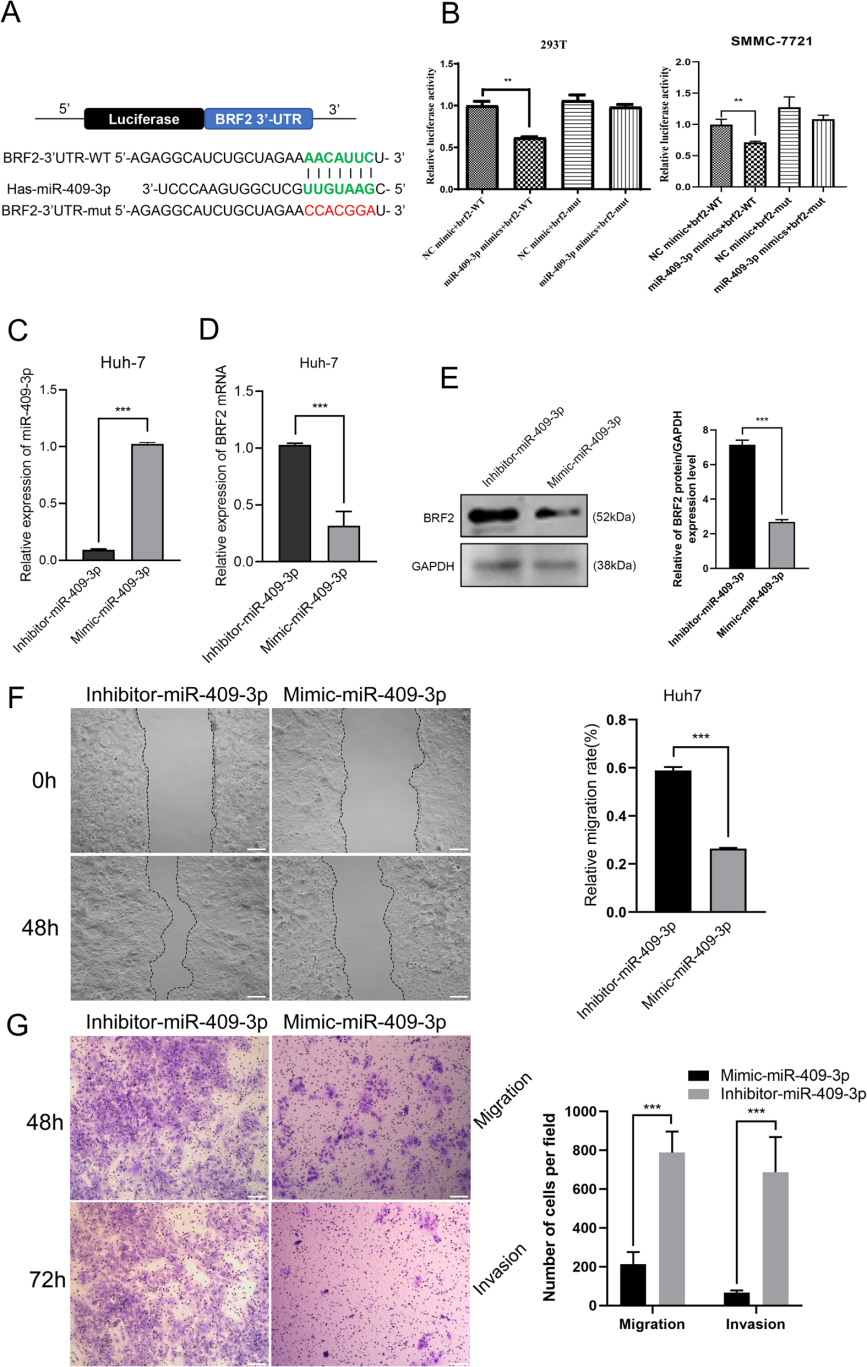
BRF2 is a direct target of miR-409-3p. **A** Predicted binding site of miR-409-3p in the BRF2 3′ UTR according to TargetScan. **B** Luciferase reporter assay detecting levels of BRF2 WT 3′ UTR (BRF2-WT) and BRF2 mutant 3′ UTR (BRF2-mut) co-transfected with either an miR-409-3p mimic or a negative control (NC) in 293 T cells and Smmc-7721cells. ∗  ∗ , P < 0.01. **C**, **D** RT-qPCR measurement of BRF2 mRNA levels after overexpression or depletion of miR-409-3p in Huh-7 cells. ∗  ∗  ∗ , P < 0.001. **E** Left: Representative western blots showing BRF2 expression in Huh-7 cells after overexpression or depletion of miR-409-3p in Huh-7 cells. Right: BRF2 levels were quantified and are given as the mean ± SD; n = 3. ∗  ∗  ∗ , P < 0.001. **F** Left: Representative images of Huh-7 cells after overexpression or depletion of miR-409-3p for a wound-healing assay. Magnification, 200 × ; scale bar, 50 µm. Right: Quantification of the wound-healing assay. n = 3. ∗  ∗  ∗ , P < 0.001. **G** Left: Images of transwell migration and invasion assays in Huh-7 cells after overexpression or depletion of miR-409-3p. Magnification, 200 × . scale bar, 50 µm. Right, Quantification of transwell migration and invasion assays. n = 3. ∗  ∗  ∗ , P < 0.001

To further interrogate the role of BRF2 in HCC, we used the RNA Target Scan database to systemically search for microRNAs that bind to BRF2 and found that miR-409-3p can bind to the 3′ untranslated region (3′ UTR) of BRF2 (Fig. 3A). To confirm this, we tested to see if miR-409-3p was able to suppress Huh-7 cell growth through decreasing BRF2 expression. We injected an miR-409-3p mimic or an miR-409-3p inhibitor into Huh-7 cells, then monitored BRF2 expression (Fig. 3C). We indeed found that miR-409-3p decreased the expression of BRF2. We verified these data using a luciferase reporter assay to measure BRF2 expression following miR-409-3p treatment. These data showed that the luciferase intensity in 293 T cells treated with miR-409-3p was significantly reduced compared with cells treated with the control (Fig. 3B). However, neither miR-409-3p overexpression nor inhibition changed the luciferase intensity in 293 T cells that were transfected with a version of BRF2 with a mutated 3′ UTR (Fig. 3B). Together, these data confirm that BRF2 is a direct target of miR-409-3p. MiR-409-3p also decreased BRF2 protein levels in Huh-7 cells (Fig. 3E).

**Fig. 4:**
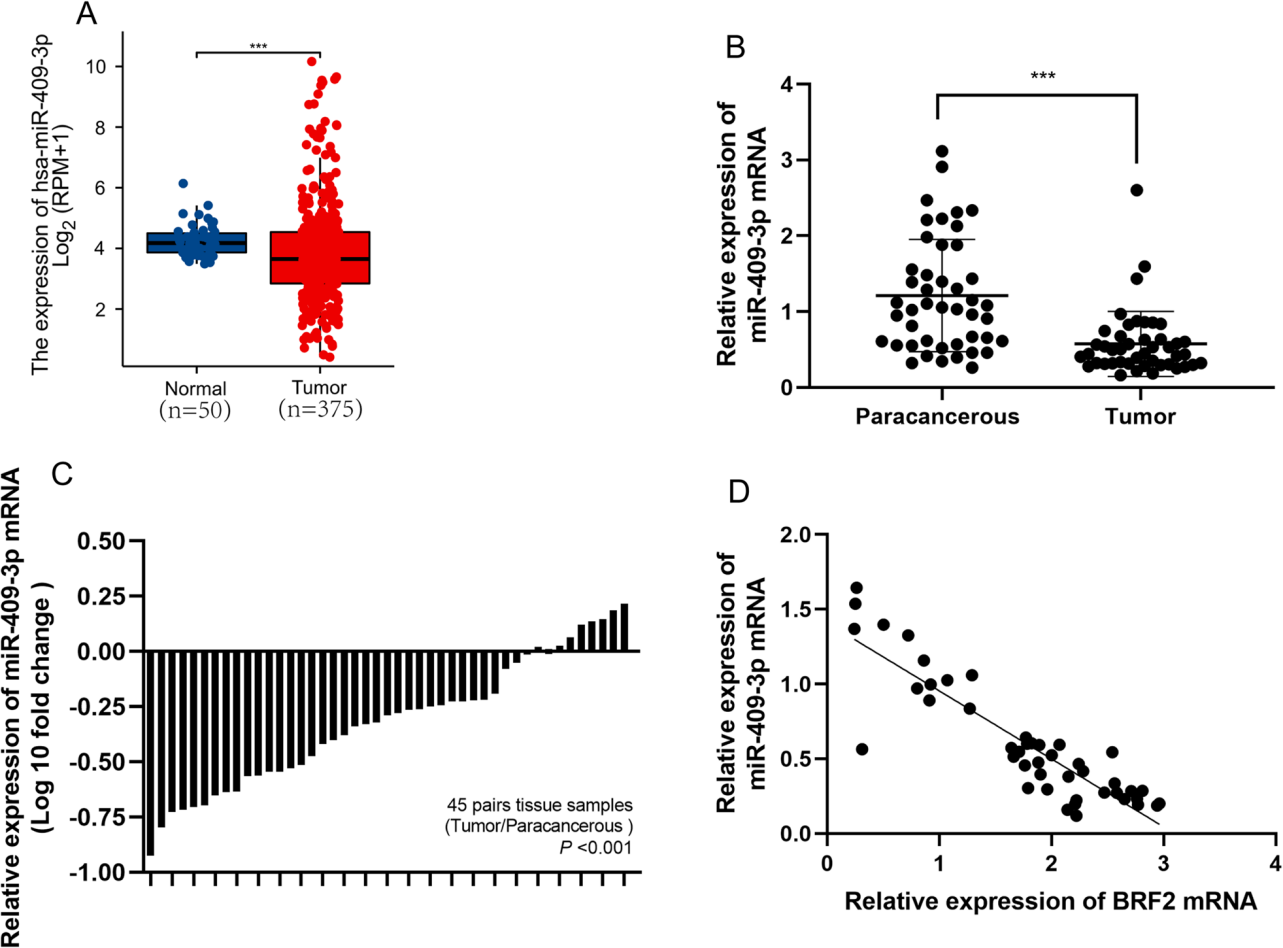
miR-409-3p is downregulated in HCC. **A** Expression of BRF2 according to TCGA and the GTEx database in liver hepatocellular cancer and normal tissues. ∗  ∗  ∗ , P < 0.001. **B** qRT-PCR data showing the expression of miR-409-3p in 45 pairs of liver hepatocellular cancer tissue and adjacent normal tissue. **C** miR-409-3p expression levels in both liver hepatocellular cancer and normal tissue. **D** Correlation between miR-409-3p mRNA levels and BRF2 levels. ***, P < 0.001

We observed that levels of miR-409-3p were decreased in liver tumor tissues compared with tumor-adjacent tissues in patient samples (Fig. 4A, B). Moreover, BRF2 expression levels were negatively correlated with miR-409-3p expression in tumors (Fig. 4C). Together, these results suggest that BRF2 is a direct target of downregulation by miR-409-3p.

### MiR-409-3p inhibits migration and invasion in vitro

To explore the function of miR-409-3p in HCC, we transfected Huh-7 cells with either an miR-409-3p mimic or an miR-409-3p inhibitor. The migration and invasion of HCC cells was then measured by wound‑healing and transwell assays. We observed that the relative migration rate of the cells treated with the miR-409-3p mimic was significantly decreased compared with cells treated with the miR-409-3p inhibitor (Fig. 3F). Furthermore, the results revealed that invasive ability of Huh-7 cells transfected with the miR-409-3p mimic was considerably lower than those treated with the miR-409-3p inhibitor (Fig. 3G).

### BRF2 promotes metastasis in vivo

**Fig. 5:**
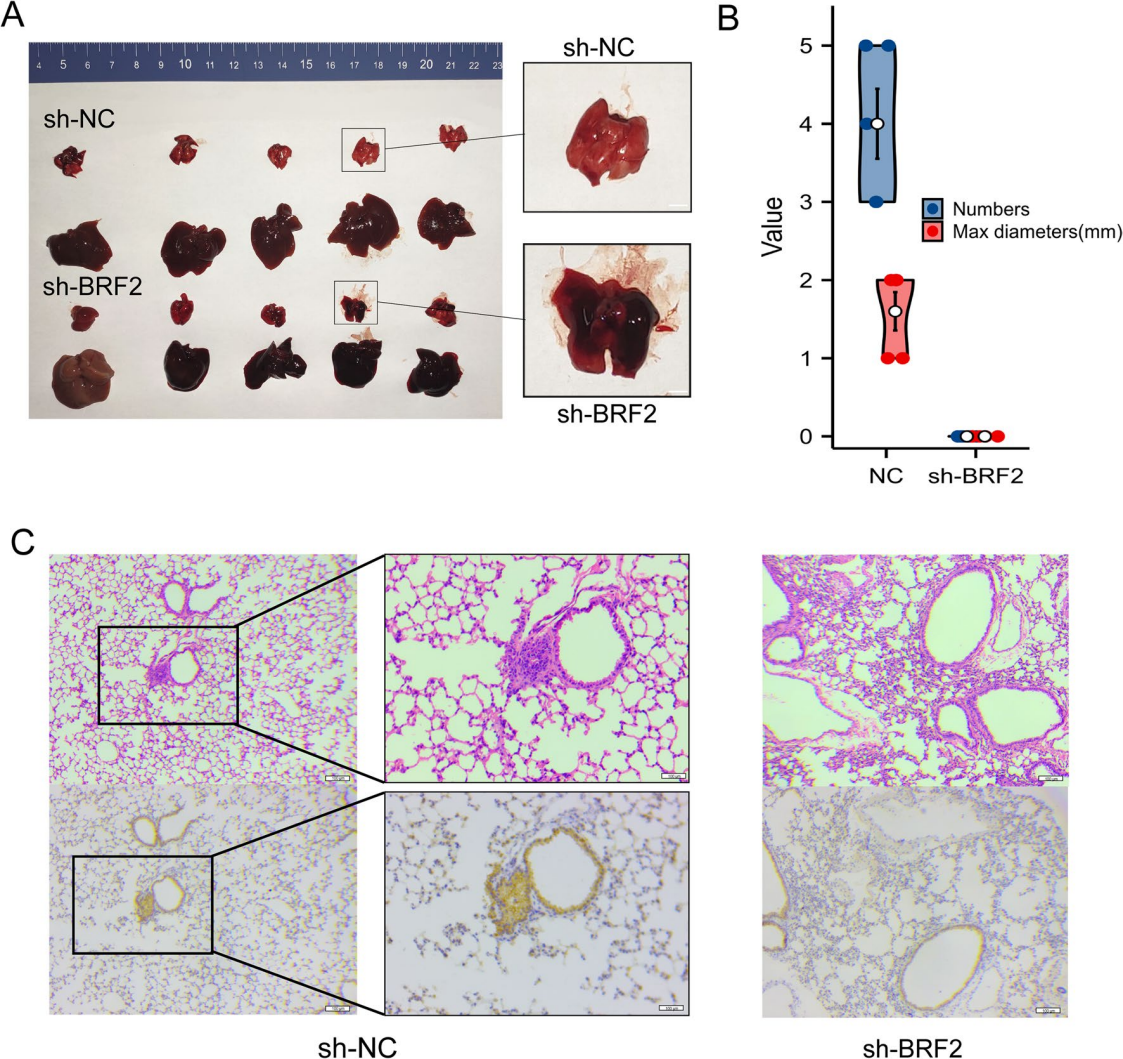
BRF2 knockdown inhibits lung metastasis of Huh-7 cells in vivo. **A** A representative photo of metastatic tumor nodules in the lungs of a nude mouse after injection with Huh-7 cells that had been treated with shRNA against BRF2 (sh-BRF2) or a negative control (sh-NC). n = 5. **B** Statistics of the nude mice with lung metastases. **C** Representative photos of metastatic tumor nodules in the lung tissue of nude mice which injected with sh-NC (left) and sh-BRF2-treated (right) Huh-7 cells after staining with hematoxylin and eosin (H&E) and IHC staining Scale bar, 200 µm

To examine the effect of BRF2 on HCC, we looked at the pulmonary metastasis potential by observing the formation of lung metastatic nodules in nude mice. We injected nude mice in the lateral tail vein with Huh-7 cells that were transfected with either sh-NC or sh-BRF2, then sacrificed the animals after six weeks. The lungs were then dissected, fixed, and stained with H&E to visualize nodules under a microscope. We found that all of the mice injected with control-treated cells had developed lung and liver metastases, while none of the mice injected with BRF2-depleted cells had developed tumors (Fig. 5).

### BRF2 exerts its tumor-promoting functions via the Wnt/β-catenin pathway in HCC

**Fig. 6:**
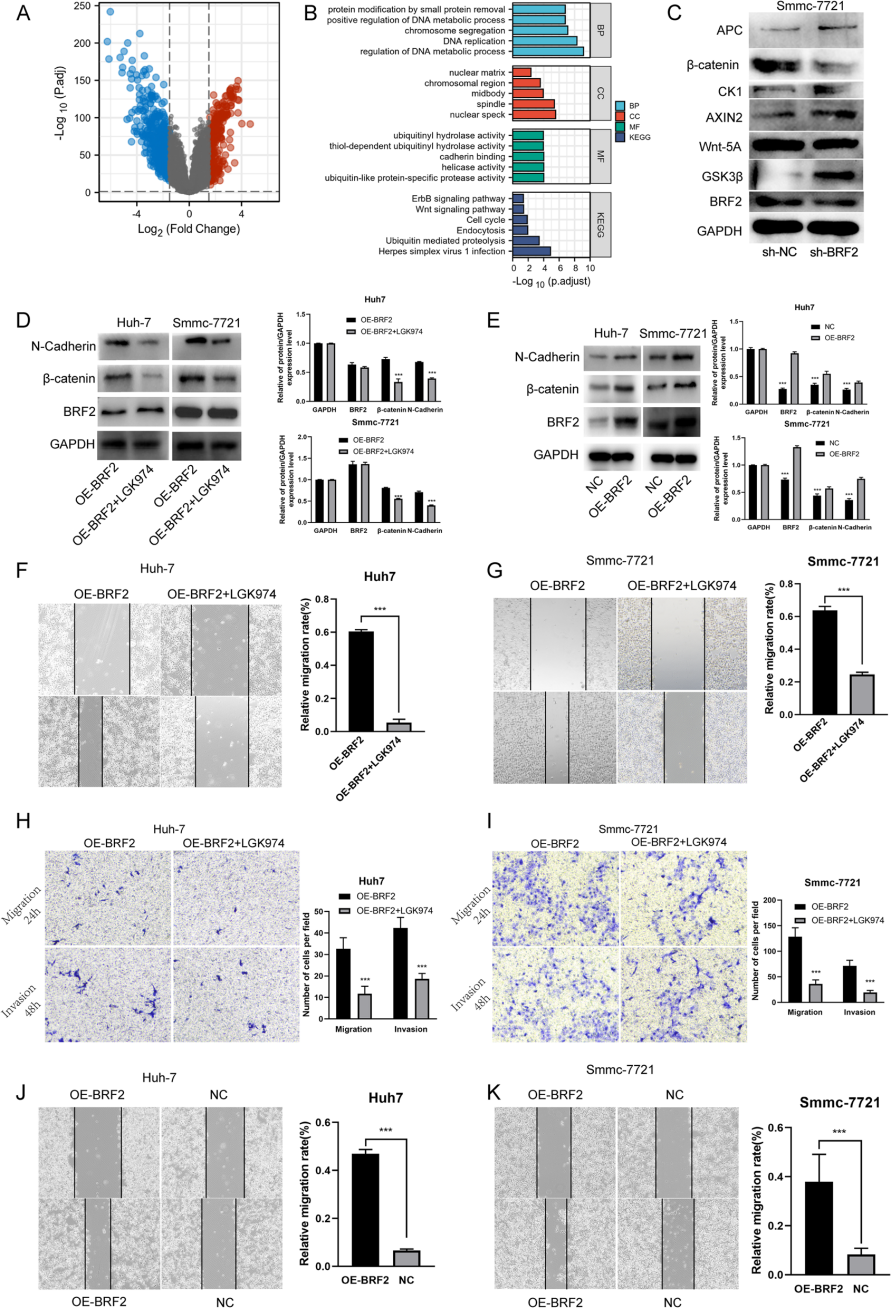
BRF2 knockdown changes key proteins within the Wnt/β-catenin pathway. Bioinformatic analysis indicates that the Wnt/β-catenin pathway may be a downstream signaling pathway of BRF2. **A** Volcano plot of single gene difference analysis according to differential BRF2 expression according to TCGA liver hepatocellular carcinoma (LIHC) database. Fold change ≥ 1.5; P < 0.05. **B** Representative graphs of KEGG/GO pathway analysis for the differentially expressed genes in different BRF2 expression groups. **C** Left: Western blots were used to verify the expression of key proteins in the downstream pathway after knockdown of BRF2. Right: Quantitative analysis of key proteins. ***, P < 0.001. **D** Representative western blot data showing N-cadherin and β-catenin in Huh-7 cells and Smmc-7721 cells treated by OE-BRF2 and NC. **E** Representative western blot data showing N-cadherin and β-catenin in Huh-7 cells and Smmc-7721 cells after infection with OE-BRF2 and OE-BRF2 + LGK974. **F**, **G** Invasion and migration of Huh-7 cells and Smmc-7721 cells treated by OE-BRF2 and OE-BRF2 + LGK974 were measured by wound-healing assay. Left: Representative images at 100 × magnification; scale bar, 100 µm. Right: Quantification of wound-healing assay. ∗  ∗  ∗ , P < 0.001, OE-BRF2 vs OE-BRF2 + LGK974 group. **H**, **I** Invasion and migration of Huh-7 cells and Smmc-7721 cells treated by OE-BRF2 and OE-BRF2 + LGK974 were measured by transwell migration and invasion assays. Left: Representative images at 100 × magnification; scale bar, 100 µm. Right: Quantification of transwell migration and invasion assays. ∗  ∗  ∗ , P < 0.001, OE-BRF2 vs OE-BRF2 + LGK974 group. **J**, **K** Left: Representative images of wound-healing assay in Huh-7 cells and Smmc-7721 cells treated by OE-BRF2 and NC. Magnification, 100 × ; scale bar, 50 µm. Right, Quantification of wound-healing assay. n = 3. ∗ ∗  ∗ , P < 0.001. OE-BRF2 vs NC group

To look at the possible roles of BRF2, we performed a bioinformatic analysis of the liver hepatocellular carcinoma (LIHC) RNAseq dataset in the Cancer Genome Atlas (TGCA) database. We divided the data into high- and low-expressing groups according to the median expression of BRF2, and identified a total of 2583 genes that showed statistically significant differences between the two groups. We then selected genes with a fold change ≥ 1.5 and a P value < 0.05, resulting in a dataset of 253 genes in the high BRF2-expressing population and 524 genes in the low BRF2-expressing population (Fig. 6A). Our next aim was to examine the potential biological processes and pathways that underlie these differentially expressed genes. We used R software (version 3.6.3), org.Hs.eg.db package (version 3.10.0), and cluster Profiler package (version 3.14.3) to perform a Kyoto Encyclopedia of Genes and Genomes (KEGG)/Gene Ontology (GO) analysis, which identified 21 significant KEGG/GO terms, including the regulation of DNA metabolic process, nuclear speck, specific protease activity, and Herpes simplex virus 1 infection (Fig. 6B). The analysis also identified the Wnt/β-catenin pathway, which plays a regulatory role in tumors. We then validated these results by western blot and found that the levels of β-catenin and Wnt-5A were all downregulated following BRF2 knockdown. And APC, GSK3β and CK1 were upregulated after BRF2 knockdown (Fig. 6C).Furthermore, overexpress BRF2 promote HCC cells migration and invasion. And then silence β-catenin could block this effect (Fig. 6D–K).

## Discussion

Hepatocellular carcinoma (HCC) is the second most lethal cancer globally [15]. Although new therapeutic advances have improved survival rates, the long-term prognosis is still poor. It is therefore critical to enhance our understanding of the disease pathogenesis and to identify new molecular targets. Many studies have shown BRF2 to be an oncogene in numerous solid tumors, including lung, gastric, and esophageal cancers, and its expression has been shown to correlate with worse clinical outcomes [16–18]. BRF2 is frequently activated during the invasion stage of lung squamous cell carcinoma [14]. BRF2 has also been recognized as an oncogenic driver in breast cancer [19]. These discoveries reveal that BRF2 plays a critical role in various tumors, and is closely linked to the invasion and migration of cancer cells [20]. In this study, we found that expression of BRF2 was higher in liver tumor tissues and cancer cells compared with normal adjacent tissues and noncancerous cells. Moreover, depleting BRF2 in Huh-7 cells increased expression of E‑cadherin while decreasing expression of N‑cadherin, both of which play critical roles in modulating signal transduction pathways for cell proliferation, cell migration, and invasion. BRF2 depletion further prevented the migration and invasion of Huh-7 cells in vitro and in vivo, revealing that BRF2 acts to promote the invasion and migration of HCC cells, potentially through modulating the epithelial-mesenchymal transition (Additional file 1).

Many studies have demonstrated that the level of the miRNA miR-409-3p is downregulated in many metastatic cancers, while high levels of miR-409-3p inhibited metastasis in gastric cancer [21–25], prostate cancer [26], bladder cancer [10], osteosarcoma [27], cervical cancer [28], lung cancer [29], breast cancer [30], and colorectal cancer. These results are consistent with the findings presented in this study showing that miR-409-3p expression was reduced in HCC tissues, and that a miR-409-3p mimic was able to reduce liver cancer cell metastasis and invasion. Importantly, we determined that miR-409-3p is able to bind to the 3′ untranslated region of BRF2 to downregulate its expression. We therefore determined that BRF2 is a target of miR-409-3p in HCC, suggesting a unique function in HCC cell migration and invasion (Additional file 2).

Tumor development is often regulated by the biochemical signaling pathways of tumor cells, such as the PI3K/AKT pathway [31, 32], the MAPK/ERK pathway [33], and the Wnt/β-catenin pathway [34, 35]. Bioinformatic analysis in combination with results from western blots indicated that BRF2 acts to modulate the invasion and metastasis of HCC cells through the Wnt pathway. We calculated the correlation between BRF2 levels and the levels of key factors within the Wnt/β-catenin signaling pathway from the LIHC RNAseq dataset from TGCA database using the starBase online platform and found significant positive correlation. Further rescue experiment which overexpress BRF2 and silence β-catenin confirm this conclusion in molecular level. (Fig. 6).

In conclusion, this study showed that BRF2 is regulated by the microRNA miR-409-3p, and that it promotes invasion and metastasis through the Wnt/β-catenin signaling pathway in HCC. Our work therefore provides further insights into the mechanism of HCC, and opens up possibilities for targeting BRF2 or miR-409-3p as therapeutic strategies against HCC.

## Supplementary Information

**Additional file 1: Figure S1.** BRF2 knockdown inhibits the invasion and metastasis of liver cancer SMMC-7721 cells. (A) Representative images of SMMC-7721 cells after lentiviral infection with si-BRF2 and si-NC was measured by wound healing assay. Magnification, x100; scale bar, 100 µm. （B）Quantification of wound healing assay. WT, Wild type. ∗∗∗, P < 0.001. sh-NC vs sh-BRF2 group. (C)Representative images of transwell migration and invasion assays in SMMC-7721 cells after lentiviral infection with si-BRF2 and si-NC. Magnification, x200. scale bar, 50 µm. (D)Quantification of transwell migration and invasion assays. n=3. WT, Wild type. ∗∗∗, P < 0.001.si-BRF2 vs si-NC group.

**Additional file 2: Table S1.**   Nodules numbers and size in lung metastasis tumor.

## Data Availability

The data during and/or analyzed during the current study available from the corresponding author on reasonable request.
