# Supplementary figures and images for: BRF2 is mediated by microRNA-409-3p and promotes invasion and metastasis of HCC through the Wnt/β-catenin pathway
Source: Cancer Cell Int. 2023 Mar 16;23:46. doi: 10.1186/s12935-023-02893-y (PMC10018885; doi:10.1186/s12935-023-02893-y)

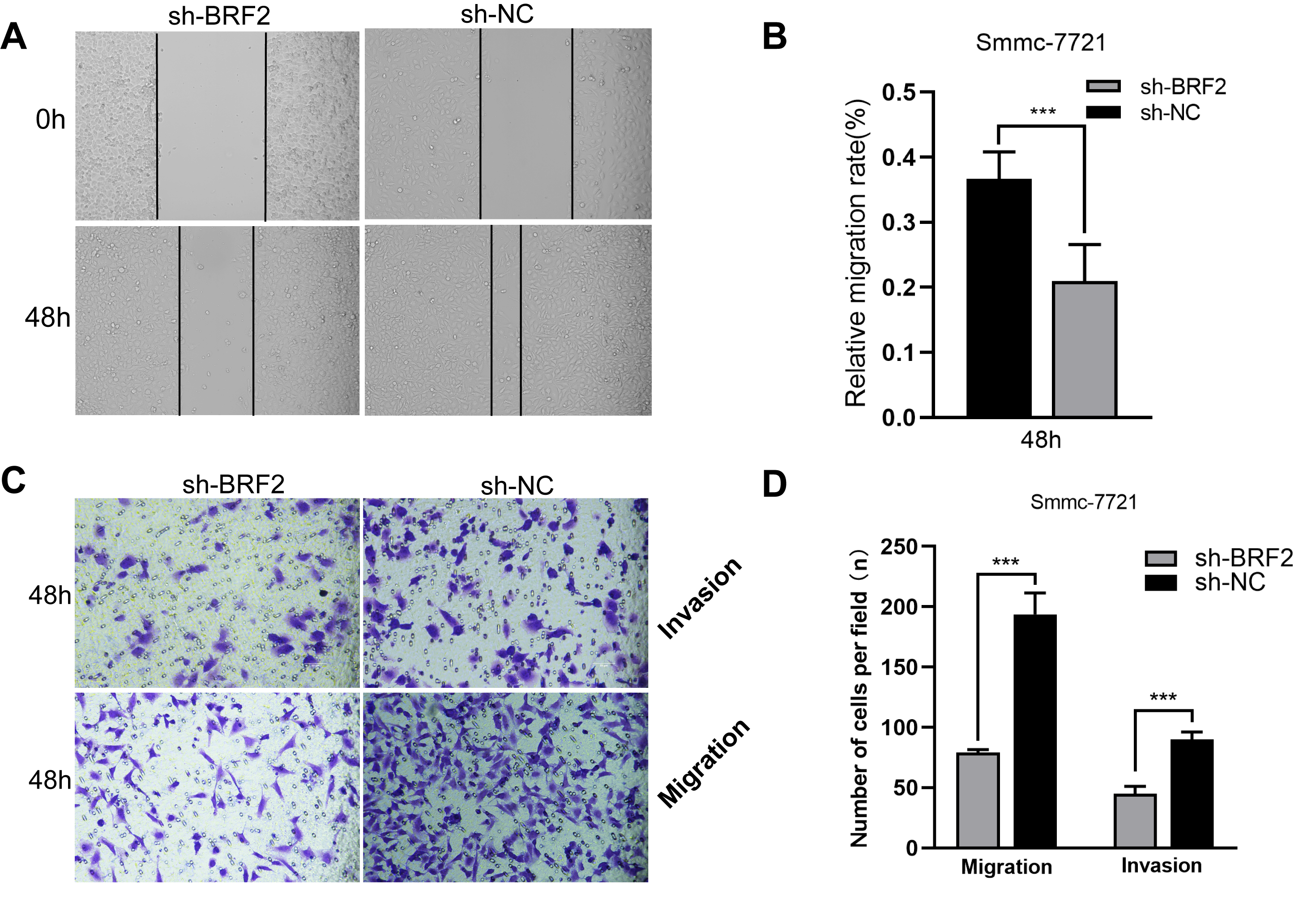

Supplement: Supplementary file 1 — Additional file 1: Figure S1. BRF2 knockdown inhibits the invasion and metastasis of liver cancer SMMC-7721 cells. (A) Representative images of SMMC-7721 cells after lentiviral infection with si-BRF2 and si-NC was measured by wound healing assay. Magnification, x100; scale bar, 100 µm. （B）Quantification of wound healing assay. WT, Wild type. ∗∗∗, P < 0.001. sh-NC vs sh-BRF2 group. (C)Representative images of transwell migration and invasion assays in SMMC-7721 cells after lentiviral infection with si-BRF2 and si-NC. Magnification, x200. scale bar, 50 µm. (D)Quantification of transwell migration and invasion assays. n=3. WT, Wild type. ∗∗∗, P < 0.001.si-BRF2 vs si-NC group. [file 12935_2023_2893_MOESM1_ESM.tif]

Nodules numbers and size in lung metastasis tumor


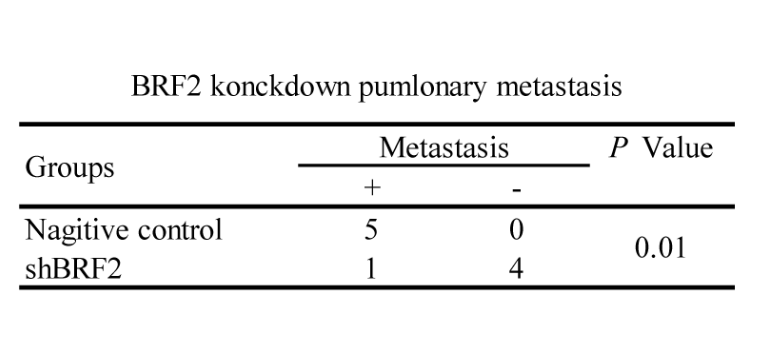


­

Supplement: Supplementary file 2 — Additional file 2: Table S1. Nodules numbers and size in lung metastasis tumor. [file 12935_2023_2893_MOESM2_ESM.docx]
